# Feasibility study of defecation studied with a wireless Fecobionics probe in normal subjects

**DOI:** 10.14814/phy2.15338

**Published:** 2022-06-03

**Authors:** Hans Gregersen, Yanmin Wang, Fred Field, Mengjun Wang, Kar Man Lo, Xiaomei Guo, William Combs, Ghassan S. Kassab

**Affiliations:** ^1^ California Medical Innovations Institute San Diego California USA; ^2^ 3DT San Diego California USA

**Keywords:** anal sphincter relaxation, anorectal physiology, bionics, defecation, fecobionics, rectoanal pressure gradient RAPG

## Abstract

Several technologies have been developed for assessing anorectal function including the act of defecation. We used a new prototype of the Fecobionics technology, a multi‐sensor simulated feces, to visualize defecatory patterns and introduced new metrics for anorectal physiology assessment in normal subjects. Fourteen subjects with normal fecal incontinence and constipation questionnaire scores were studied. The 10‐cm‐long Fecobionics device provided measurements of axial pressures, orientation, bending, and shape. The Fecobionics bag was distended to the urge‐to‐defecate level inside rectum where after the subjects were asked to evacuate. Physiological evacuation parameters were assessed. Special attention was paid to the Fecobionics rectoanal pressure gradient (F‐RAPG) during evacuation. Anorectal manometry (ARM) and balloon expulsion test (BET) were done as references. The user interface displayed the fine coordination between pressures, orientation, bending angle, and shape. The pressures showed that Fecobionics was expelled in 11.5 s (quartiles 7.5 and 18.8s), which was shorter than the subjectively reported expulsion time of the BET balloon. Six subjects did not expel the BET balloon within 2 min. The F‐RAPG was 101 (79–131) cmH_2_O, whereas the ARM‐RAPG was −28 (−5 to −47) cmH_2_0 (*p* < 0.001). There was no association between the two RAPGs (r^2^ = 0.19). Fecobionics showed paradoxical contractions in one subject (7%) compared to 12 subjects with ARM (86%). Fecobionics obtained novel physiological data. Defecatory patterns and data are reported and can be used to guide larger‐scale studies in normal subjects and patients with defecatory disorders. In accordance with other studies, this Fecobionics study questions the value of the ARM‐RAPG.

## Graphical abstract

Fecobionics was further developed into a wireless device that integrated measurements of axial pressures, orientation, bending, and shape changes. Pressure‐bending‐shape integrated patterns are presented from normal subjects. Fecobionics data differed significantly from BET expulsion duration and ARM‐RAPG data.

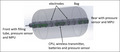

## 1 INTRODUCTION

Defecation is a complex physiological process through which stools are eliminated via the anus (Gibbons, 1988; Gregersen & Christensen, 2016; Suares & Ford, 2011). Defecation is initiated by an urge‐to‐defecate predominantly resulting from filling of stool in rectum. During evacuation, the abdominal pressure increases, the anal sphincter relaxes, and the anorectal angle straightens. If anorectal organs and function get disturbed, defecatory disorders with symptoms such as pain, fecal incontinence (FI), and chronic constipation (CC) may develop (Rao et al., 2016). Defecatory disorders affect 25% of the population and pose a major health care burden but are not optimally recognized and treated (Rao et al., 2016; Suares & Ford, 2011). Despite the use of several technologies for studying anorectal function in physiology labs, there is a need for physiologically relevant and easy‐to‐use diagnostic tests for identifying underlying mechanisms for normal function and defecatory disorders.

The standard technologies for studying anorectal function are high‐resolution anorectal manometry (ARM), balloon expulsion test (BET), and defecography (Bharucha, 2006; Carrington et al., 2019; Chiarioni et al., 2014; Rao et al., 2016; Tirumanisett et al., 2018; Van Koughnett & Silva, 2013). Fecobionics was developed for studying anorectal physiology in a more physiological and integrated way (hence the name Feco‐bionics) since BET merely provides data on the time it takes to expel the balloon, ARM is not done during defecation, and pressure is not measured during defecography. Despite their evident clinical applications, for instance BET is a simple and useful screening test to identify constipated patients (Minguiz et al., 2004), disagreement exists between the results of various anorectal tests, they do not correlate well with symptoms and treatment outcomes, and dyssynergic patterns are frequently found in normal subjects (Coss‐Adame et al., 2015; Grossi et al., 2016; Palit et al., 2016; Rao et al., 2016). However, previous versions of Fecobionics merely measured pressures, were tethered (wired), and did not have an integrated graphical user interface.

From a mechanistic point of view, the pressure difference between the rectum and anal canal is an important factor for defecation and the continence mechanisms (Gregersen, 2002; Gregersen & Christensen, 2016; Li et al., 2013; Noelting et al., 2012). Therefore, parameters like the rectoanal pressure gradient (RAPG) have been developed for ARM studies but the value of the RAPG has been disputed (Grossi et al., 2016). We designed Fecobionics to integrate important functional measures in a single test (Chen et al., 2020, 2021a, 2021b; Gregersen, 2020; Gregersen et al., 2019, 2018; Gregersen, Chen, et al., 2021; Gregersen, Sun, et al., 2021; Gregersen, Wang, et al., 2021; Kassab et al., 2021; Sun et al., 2019, 2020; Zhuang et al., 2021). The foundation was laid with the device that measured axial pressures in contrast to the radial pressures measured by ARM. Technological validation has been published (Sun et al., 2019, 2020). Among other parameters, it was demonstrated that defecation can be divided into five phases (Gregersen et al., 2018) and “preload‐afterload diagrams are an informative way to demonstrate the dynamics of defecation (Chen et al., 2020, 2021a; Gregersen, 2020; Gregersen et al., 2019; Gregersen, Sun, et al., 2021; Kassab et al., 2021). Other studies have been done on patients with FI including patients with low anterior resection syndrome (LARS) and CC including obstructed defecators (Chen et al., 2021b; Gregersen et al., 2020; Gregersen, Chen, et al., 2021). Furthermore, pilot studies have been done with an early stage integrated Fecobionics prototype in colon and anorectum (Gregersen, 2020; Gregersen, Sun, et al., 2021; Gregersen, Wang, et al., 2021; Kassab et al., 2021; Sun et al., 2020).

This study represents a significant advancement in technology development for obtaining a mechanistic understanding of defecation. The Fecobionics device was developed into an integrated wireless test with the graphical user interface showing simultaneous recordings of pressures, orientation, bending, cross‐sectional areas, and shape. This provides unprecedented possibilities since these measures are obtained simultaneously in a single test.

The aim of this study was to evaluate the feasibility and performance of the new integrated Fecobionics for the assessment of defecatory patterns and new defecation metrics in normal subjects. We provide detailed descriptions of defecatory patterns in normal subjects to serve as a reference for larger trials in normal subjects and future clinical studies. Furthermore, we emphasize the expulsion duration and the Fecobionics rectoanal pressure gradient (F‐RAPG) during evacuation, and compare these parameters between technologies. It was not an aim to compare with the original Fecobionics device since the basic device design was not changed and studies were done on different populations.

## 2 MATERIALS AND METHODS

### 2.1 Subjects

Presumed normal subjects were invited to participate in this exploratory study through advertisement at the California Medical Innovations Institute in San Diego, California. The lower age limit was 18 years with no upper age limit imposed. If deemed “normal” during a phone interview, where we asked about medications (use of GI medications were not allowed), abdominal surgery, and chronic diseases, subjects were asked to visit the CALMI2 anorectal function laboratory for further interview, questionnaires, and anorectal physiology testing. Data were obtained on gender, age, symptoms, health status, disease conditions, and previous treatments. FI and CC questionnaires data were obtained (Agachan et al., 1996; Rockwood, 2004). All subjects with FI and CC scores that exceeded the limit for normality were excluded (*n* = 6). The subjects were considered normal if FI and CC questionnaire scores <5 (Agachan et al., 1996) and <8 (Rockwood, 2004). Fourteen subjects were enrolled and studied. All 14 subjects had the tests completed. The protocol was approved by the IntegReview IRB (reference no. CALM‐CLIN‐2019–01). The trial was registered at www.clinicaltrials.gov with identifier: NCT04765138.

### 2.2 Procedures and testing techniques

The Fecobionics and ARM‐BET tests were done in random order on the same day using a predefined randomization scheme. The subjects were asked to empty their rectum if they were able to prior to the experiments. Enema was not used to make the test as natural as possible. Digital anorectal exploration was done prior to insertion of the devices to assess anal tone and verify that the lower rectum was empty.

**FIGURE 1:**
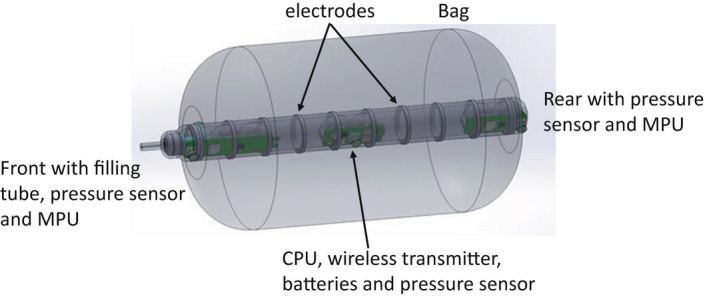
Schematic of the wireless Fecobionics device. The device contains 3 pressure sensors placed at the front, rear, and inside the bag. Furthermore, it contains two motion processor units (MPUs consisting of 3D gyroscope, accelerometer, and magnetometer) for the determination of orientation and bending (the anorectal angle). The core has multiple electrodes that utilize the impedance planimetric principle to determine the geometry of the bag. The core also contains the central processing unit, wireless transmitter, and batteries. A valve and filling tube system is attached at the front. The detachment system secures that the device is untethered after bag filling (not shown).

The basic design of the previous Fecobionics prototype has been previously described (Chen et al., 2020; Gregersen, 2020; Gregersen, Sun, et al., 2021; Kassab et al., 2021; Sun et al., 2019). Previous versions recorded pressures only or did not have an integrated graphical user interface. The new integrated and wireless design used in this study is shown in Figure 1. The core of the new Fecobionics device is 10‐mm‐OD, 10‐cm‐long. The core contains pressure sensors at the front‐rear and inside the bag, two motion processing units (MPUs with gyroscopes, accelerometers, and magnetometers), electrodes for impedance planimetric measurement of seven cross‐sectional areas (CSAs), and other electronics (electronic circuit boards, wireless transmitter, and batteries) embedded in the silicone core. The 9‐axis MPU data are used to compute the orientation in 3D space and the bending of the device. The default graphical user interface shows the orientation of the motion sensors relative to the field of gravity and the angle between them. Hundred and eighty degrees indicate that the device is straight. The bending angle is 180 degrees minus the measured angle. When the device passes from rectum into the anal canal, this is a measure of the anorectal angle. The seven CSAs were used to model the shape of the bag in rectum and during passage through the anal canal during evacuation. The core has a 9 cm‐long cylindrical bag mounted for rectal distension. With the architecture, hardness shore, and the bag, Fecobionics has form and consistency that corresponds approximately to type 4 (range 3–4) on the Bristol stool form scale (Heaton et al., 1992). The range from types 3–4 is found in +60% of normal subjects (Heaton et al., 1992). The bag was connected through a detachable fill tube extending from the front of Fecobionics to a syringe containing body‐warm saline. All data were acquired at 50 Hz except the impedance data that was acquired at 20 Hz. Wireless data transmission was done to a wireless receiver unit placed next to the subject in real‐time using the ISM band (902–928 MHz).

A curtain shielded the patient during the study and the investigators left the room during the defecation. Fecobionics was manually inserted in rectum with the subject in left lateral position. The subject changed from horizontal to sitting position after the insertion and moved to the commode chair. After approximately 5 min of resting, the subjects performed maneuvers with appropriate time in between. The subjects were asked to cough twice, squeeze the anal muscle three times for 5 s, do a long squeeze, do straining for 15 s, and three push procedures for 15 s. Subsequently, the bag was distended until urge‐to‐defecate or to maximum 100 ml. The volumes at first sensation, slight sensation of urge, and at urge were noted and the subjects were asked to evacuate Fecobionics. The evacuation duration was easily determined from the recordings; that is, from pressure increased above baseline until all three pressures reached atmospheric pressure. However, to compare with BET which does not measure pressures, we also counted the time from the subjects were asked to evacuate to the act was completed.

ARM‐BET experiments were done as a reference since they are standard technologies for anorectal assessment. In general, the purpose was not to compare technologies since devices and procedures are significantly different and previous studies showed variations due to differences in technology and methodology (Chen et al., 2020, 2021a, 2021b; Gregersen, 2020; Gregersen et al., 2019, 2020; Gregersen, Chen, et al., 2021; Gregersen, Sun, et al., 2021; Kassab et al., 2021; Sun et al., 2020). ARM was conducted with a standard high‐resolution anorectal catheter (Manoscan, Medtronic Inc., USA). The manufacturer's instructions for use were followed. It was inserted with the subjects lying in the left lateral position with bended hip and knees. The bag was placed in the rectum and pressures were measured at 0.5 cm distance in the anal canal. Resting and squeeze anal pressures, the recto‐anal inhibitory reflex (RAIR), first sensation, urge, and maximum tolerable volumes and the RAPG (named the rectoanal pressure differential in the Manoscan system) were extracted from the ARM reports from the Manoscan system. For BET, the SR1B single‐use anorectal balloon catheter was used. Instructions for use by the manufacturer was followed (Mui Scientific, Canada). The expulsion duration for the 50 ml fluid‐filled balloon catheter was measured. The BET evacuation was done on the commode chair.

### 2.3 Data analysis and statistics

Multiple parameters were calculated including the questionnaire scores, duration of the whole experiment, expulsion duration, bending angles, pressure amplitudes, expulsion velocity, and preload‐afterload pressure plots (Chen et al., 2021a, 2021b; Gregersen, 2020; Gregersen et al., 2019; Gregersen, Chen, et al., 2021; Gregersen, Sun, et al., 2021; Kassab et al., 2021; Takeuchi et al., 2003). The expulsion velocity was derived from the color (diameter) topographies when the device was half evacuated. The preload‐afterload diagram plots the front pressure as a function of the rear pressure. It is a novel way to express Fecobionics data (Gregersen, 2020; Gregersen, Sun, et al., 2021; Kassab et al., 2021), which relates to the RAPG. For defecation, the rectum or abdominal muscle contractions generate the preload whereas the afterload is due to anal resistance. Fecobionics measures the preload and afterload with the rear and front pressure sensors. Paradoxical contractions were defined as a sudden increase in the front pressure (active anal contraction above the rear and bag pressures) during the device evacuation.

Due to the relatively low number of subjects, the experimental data were generally considered non‐parametric and consequently, the median and quartiles were computed. For demographic data and study duration, we used parametric statistics. Data for the repeated coughs, anal squeezes, and pushes were averaged, respectively. *t* test, Wilcoxon Signed Rank test, and Mann–Whitney *U* test were used for studying differences for unpaired and paired data. Pearson´s correlation was used for the analysis of association of data obtained with the technologies employed. Scatter plots with regression lines and Bland Altman plots were generated for comparison between two methods. Results were considered statistically significant when *p* < 0.05 (2‐tailed).

## 3 RESULTS

The 14 subjects studied were seven females and seven males. The age, height, weight, and BMI were 40.5 ± 4.1 years, 171.8 ± 2.1 cm, 79.9 ± 5.3 kg, and 27.2 ± 2.0 kg/m^2^, respectively. The FI and CC scores were 0 (0–0) and 3 (2–5).

The Fecobionics studies lasted 25.3 ± 1.0 min, which was similar to ARM‐BET (24.2 ± 0.9 min). Basic ARM‐BET data are provided in Table 1. Relevant ARM‐BET data for comparison with Fecobionics are provided in the text below.

**TABLE 1:** Anorectal manometry (ARM)–balloon expulsion test (BET) basic data

BET expulsion duration	66.0 (40.3–120) s^a^
Mean resting sphincter pressure	107.1 (84.3–114.3) cmH_2_0
Maximum resting sphincter pressure	118.8 (88.6–129.1) cmH_2_0
Maximum squeeze sphincter pressure	269.4 (233.3–347.8) cmH_2_0
Push rectal pressure	74.2 (61.2–80.3) cmH_2_0
Push residual anal pressure	104.0 (66.5–122.0) cmH_2_0
RAPG	−28 (−5 to −47) cmH_2_0
First sensation during distension	47.5 (24.3−58.8) cmH_2_0
Urge during distension	72.5 (60.0–115.0) cmH_2_0
Discomfort during distension	130.0 (120.0–166.3) cmH_2_0
Paradoxical contraction^b^	12/14 (86%)

Notes

ARM‐BET means anorectal manometry and balloon expulsion test. RAPG is the rectoanal pressure gradient.

^a^ Six subjects used 2 min or longer to evacuate BET.

^b^ Paradoxical contraction is defined as the number of subjects with negative RAPG, that is, where the anal pressure exceeded the rectal pressure during push.

Incidences or adverse effects were not reported during the studies. Specifically, the Fecobionics procedures (insertions, maneuvers, bag distension, and evacuation) did not cause pain, other unexpected symptoms or bleeding. No device related malfunctions were detected during the studies as well as during inspection and test after the experiments. During experiments on two subjects, the front pressure showed faulty or missing data. Hence, in these cases, the front pressure and the derived F‐RAPG were excluded. The average data loss was less than 1%.

**FIGURE 2:**
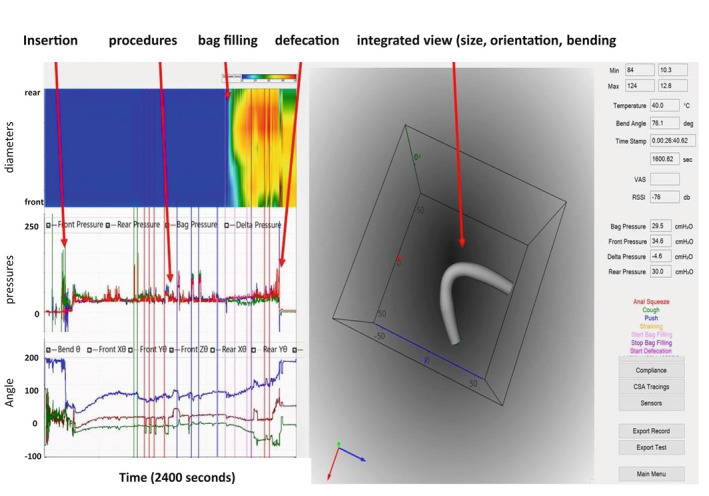
The Fecobionics graphical user interface showing pressures, orientation, bending, and shape. Training exercises (coughs, anal squeezes, push, and straining procedures) were conducted from 800 to 500 s (see Figure 3). This was followed by filling of the bag and evacuation. In the diameter color topography, blue to red indicate increasing diameter from 10 to 40 mm. In the pressure plot, the rear, bag, and front pressures are indicated with green, red, and blue colors, respectively. In the lower‐left plot, the bending angle is shown in blue color. The green and brown tracings are the orientation of the two MPUs relative to the field of gravity.

Figure 2 shows the graphical user interface from a representative subject for an entire experiment with color topography data of the diameters, the three pressures, orientation, bending angle, and shape.

### 3.1 Pre‐distension maneuvers

Coughing induced simultaneous median pressure increase in all channels to around 170–190 cmH_2_O; for example, the bag pressure was 170 (142–210) cmH_2_O. No difference was found between the three pressures (*p* > 0.5) and the coughs did not change the bending angle (approximately 140°, *p* > 0.05).

The anal squeeze pressure measured with the front sensor was 50 (44–63) cmH_2_O. The bending angle change was minimal from 139° (112–151°) to 128 (104–141°) before and during the anal squeezes (*p* > 0.05).

**FIGURE 3:**
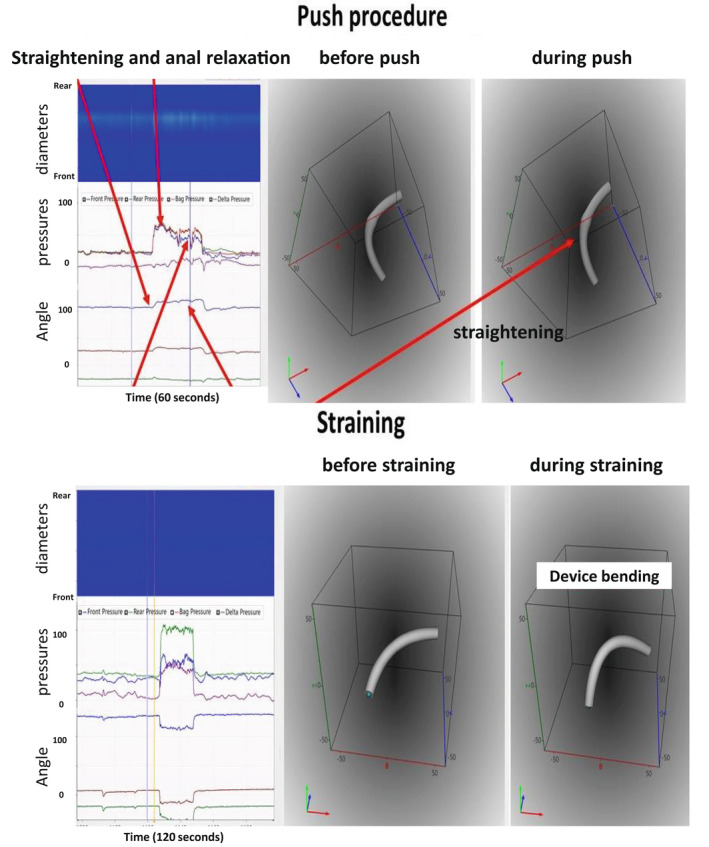
Top: Correctly done push procedure with anal relaxation and device straightening. Bottom: Straining procedure showing device bending despite less increase in front pressure (blue). Other pressures shown are the rear pressure (green) and the pressure difference between the rear and front pressure (violet). The changes in bending angle during the two procedures were as expected, that is, less bend during push and more bend during straining. In the diameter color topography, blue to red indicate increasing diameter from 10–40 mm. In the pressure plot, the rear, bag, and front pressures are indicated with green, red, and blue colors, respectively. In the lower‐left plot, the bending angle is shown in blue color. The green and brown tracings are the orientation of the two MPUs relative to the field of gravity.

The push procedure increased pressures in the three channels to 75–90 cmH_2_O; for example, the bag pressure was 79.6 (67.1–91.1) cmH_2_O. The maximum pressure difference between the rear and front channels was 13.6 (9.2–20.1) cmH_2_O. The bending angle was 134 (111–150°) and 142 (117–155°) before and during the push procedure (*p* > 0.1). Straining increased pressures in the three channels to 80–95 cmH_2_O; for example, the bag pressure was 90.2 (82.2–108.5) cmH_2_O. The maximum pressure difference between the rear and front channels was 8.6 (4.3–18.1) cmH_2_O. The median decrease in bending angle change was small from 136 (115–144) to 133 (110–144°) during straining (*p* > 0.5). However, some subjects demonstrated clear changes in bending angle whereas others did not. The subjects, who showed changes in bending angle during straining and push procedures, had higher front pressure and lower bending angle than other subjects, which points to a more distal (anal) location of the device in anorectum. Furthermore, some subjects seem to have difficulty doing the push procedure and some reported they hold back in order not to evacuate the device. Figure 3 shows examples where simultaneous changes in pressure and bending angle were evident.

### 3.2 Bag distension

The bag was distended until the subjects felt urge‐to‐defecate. The distension resulted in bag pressure increase from 27 (19–38) to 45 (34–51) cmH_2_O (*p* < 0.01) and often in concomitant internal anal sphincter (IAS) relaxation recorded by the front pressure sensor. RAIR was observed in 9 of 13 cases. The volumes at first sensation, mild urge, and urge were 22.5 (16.5–30.0), 46.5 (32.5–59.0), and 75.0 (53–91) ml, respectively. Three subjects reached the maximum 100 ml volume. Before bag distension, the bending angle was 147 (106–150). The bag distension straightened the device to 160 (140–167), *p* < 0.01), which may be due to relocation to a more proximal location inside the rectum or to the pressure‐induced force on the organ wall.

### 3.3 Evacuation of Fecobionics

**FIGURE 4:**
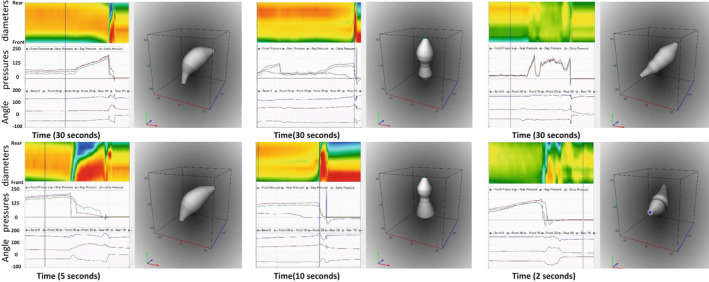
Six representations so the graphical user interface. The data are obtained from three subjects. The top views show the time from initiation of contraction to the device is evacuated. The bottom plots show subsections of the upper plots, that is, the passage of Fecobionics through the anal canal. In the diameter color topography, blue to red indicate increasing diameter from 10 to 40 mm. In the pressure plot, the rear, bag, and front pressures are indicated with green, red, and blue colors, respectively. In the lower‐left plot, the bending angle is shown in blue color. The green and brown tracings are the orientation of the two MPUs relative to the field of gravity.

**FIGURE 5:**
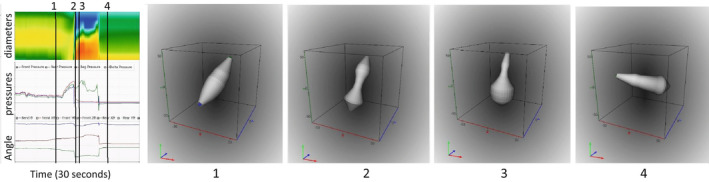
Illustration of a representative evacuation. The left diagram shows diameters color topography, pressures, orientation, and bending angle. The four vertical lines show four different stages during the device evacuation. The shape corresponding to each vertical line is shown to the right. The passage through the anal canal can clearly be observed. See also the video in the supplementary material. In the diameter color topography, blue to red indicate increasing diameter from 10 to 40 mm. In the pressure plot, the rear, bag, and front pressures are indicated with green, red, and blue colors, respectively. In the lower‐left plot, the bending angle is shown in blue color. The green and brown tracings are the orientation of the two MPUs relative to the field of gravity.

**FIGURE 6:**
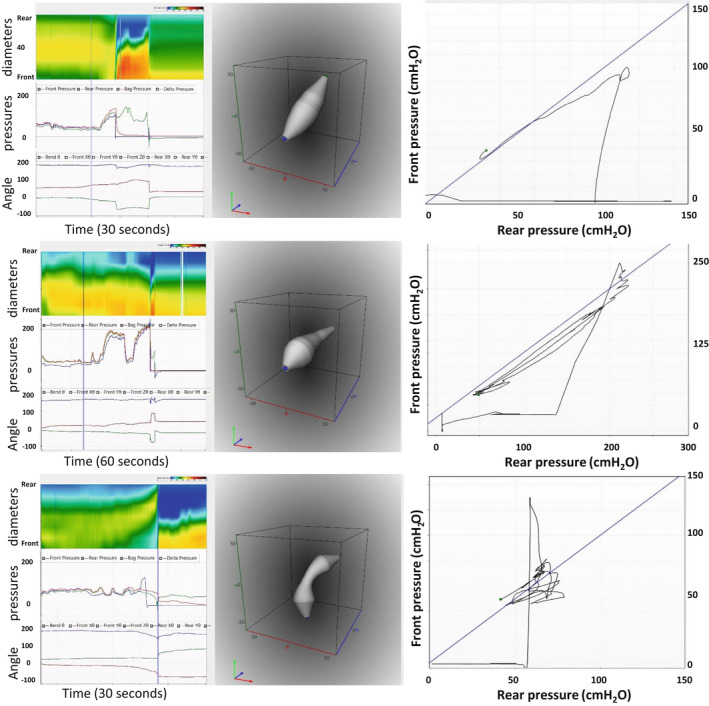
Patterns (angle, pressures, and diameter color topography) as a function of time is shown to the left. The middle diagrams are the 3D orientation and shape plots. The shape is obtained at the time point illustrated with the vertical line in the left diagrams. The right diagrams show the rear‐front pressure data and the line of unity pressure. The data are obtained from subjects who evacuated the device with one contraction (top) and two contractions (middle). The bottom figures represent the subject with a paradoxical contraction immediately before evacuation. In the diameter color topography, blue to red indicate increasing diameter from 10 to 40 mm. In the pressure plot, the rear, bag, and front pressures are indicated with green, red, and blue colors, respectively. In the lower‐left plot, the bending angle is shown with blue color. The green and brown tracings are the orientation of the two MPUs relative to the field of gravity.

After the urge‐to‐defecate level was reached, the subjects were asked to evacuate Fecobionics in privacy. Figure 4 shows representative patterns of evacuations from three subjects. Figure 5 illustrates shape changes at 4 time points in one of the subjects. A video sequence of the same is included in the Supplementary material. Figure 6 shows the defecatory patterns including rear‐front pressure plots from three subjects.

The expulsion duration was 11.5 (quartiles 7.5 and 18.8) seconds based on the pressure recordings. All subjects expelled the device within 1 min. The duration was different from the BET duration (*p* < 0.01). However, when evaluated based on the reporting by the subjects, the duration was 43 (27.8–86.3) seconds and one subject reached the 2 min limit. In this case, there was no difference between Fecobionics and BET duration (*p* > 0.01, Table 1). The expulsion duration was not associated with the urge volume (*r* = 0.15, *p* > 0.5).

Based on our definition of normal or abnormal first contraction, we found that all subjects showed a normal initial pressure profile. One subject (7%), however, had a paradoxical contraction right before the device was evacuated (Figure 6). Twelve subjects (86%) had abnormal pressure profiles during ARM push procedure (Table 1).

Seven subjects evacuated the Fecobionics device with just one contraction (Figures 5 and 6). Two, four, and one subjects used 2, 3 or 5 contractions, respectively. The typical pattern was that the pressure initially increased simultaneously in all three pressure channels but the front pressure would level off due to anal sphincter relaxation. This occurred at a front pressure of 87.0 (66.2–105.25) cmH_2_O corresponding to 50.0 (31.3–75)% of the maximum pressure during defecation. The maximum pressures during evacuation were recorded in the bag and rear pressure channels. Most often the bag pressure (126.5 (90.8–152.5) was higher than the rear pressure (116.0 (78.3–146), *p* < 0.05)). The front often recorded atmospheric pressure before the rear pressure reached maximum. The rear‐front pressure diagrams express clockwise contraction cycles (Figure 6). For those who used more than one contraction to evacuate, the repeated contractions translated the tracings downwards and, at some point, a cut‐off was reached where the anal pressure dropped quickly followed by complete evacuation of the device.

The pressure changes were accompanied by simultaneous changes in the color topographies (Figures 4, 5, 6). In some cases, the probe started to move distally when the contraction started. However, this was not always the pattern. Clearly, the movement was dependent on the difference between the front pressure and the two other pressures as well as on straightening of the device, which likely represents a relaxation of the puborectalis muscle (more obtuse anorectal angle).

**FIGURE 7:**
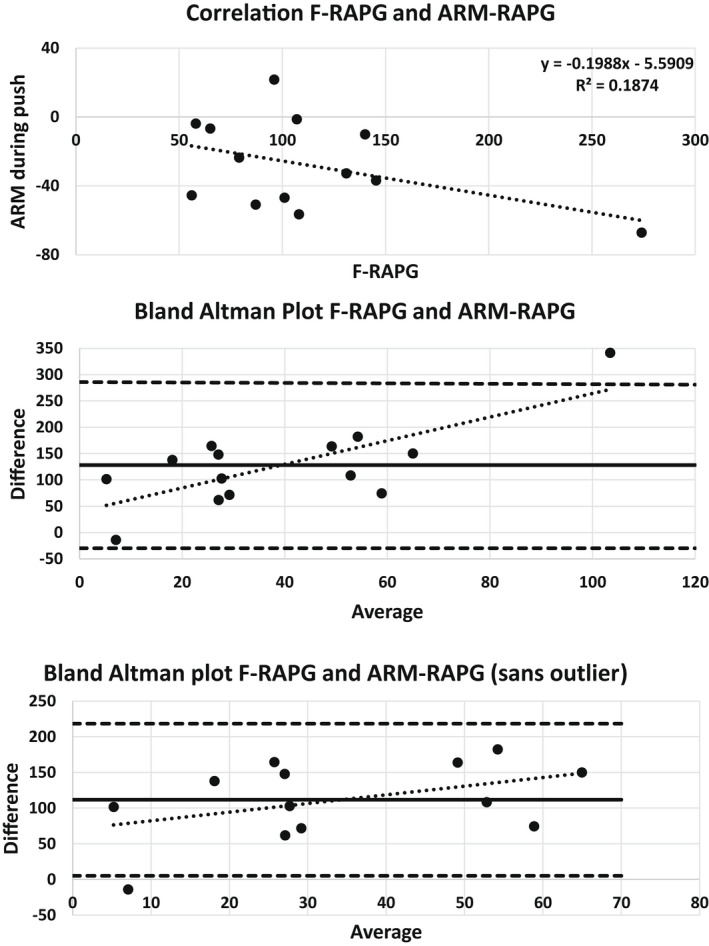
Association between the Fecobionics rectoanal pressure gradient during evacuation (F‐RAPG) and the anorectal manometry rectoanal pressure gradient during evacuation (ARM‐RAPG). The top shows the correlation plot. The middle diagram is the corresponding Bland Altman plot. The lower diagram is a Bland Altman plot where one outlier has been removed.

The maximum difference between the rear and the front pressures during evacuation (the F‐RAPG) was 101 (79–131) cmH_2_O. There was no association between the F‐RAPG and the ARM‐RAPG (*r* = 0.43, *p* > 0.1). Bland‐Altman plots showed that the bias was just within the limits and that the difference data increased with the average (Figure 7). If the outlier in the figure was removed, the bias was outside the limits (Figure 7). Furthermore, the F‐RAPG was also not associated with the Fecobionics push maximum pressure difference (*r* = 0.13, *p* > 0.4). The ARM‐RAPG and the Fecobionics push maximum pressure difference differed (*p* < 0.01) and there was no association between the data (*r* = 0.15, *p* > 0.5).

The expulsion velocity derived from the color topography plots was 30 (19–33) cm/s. The expulsion velocity was not associated with the F‐RAPG (*r* = 0.41, *p* > 0.2) or with the urge volume (*r* = 0.15, *p* > 0.5).

The front and rear had an average orientation of 50 and 20 degrees relative to the field of gravity before evacuation but some variability was encountered (Figures 2, 4, 5, 6). The orientation approached vertical (90°) when passing from the rectum into the anal canal. The device landed in almost horizontal position in the pot below the commode chair, which was confirmed by the orientation measurement. The bending angle immediately before evacuation was 164° (151–166°). During evacuation, the bending angle decreased to 145° (127–158°), which likely represents the anorectal angle.

## 4 DISCUSSION

This study represents the first human experiment with a greatly upgraded Fecobionics device. Previous prototypes were shown to be safe, were validated but only measured pressures and were wired (Sun et al., 2019, 2020). They were used in studies of normal subjects (Chen et al., 2021a; Gregersen et al., 2018, 2019; Sun et al., 2020), patients with FI (Gregersen et al.,) LARS (Chen et al., 2021b), and obstructed defecation (Gregersen, Chen, et al., 2021) as well as it has proven to be able to detect perineal descent (Zhuang et al., 2021). Fecobionics provides a new bionics tool to facilitate studies of anorectal physiology and diagnostics by integrating several current tests in wireless technology. The aim of the current study was to describe defecatory physiology and performance to serve as a reference for future larger scale studies. We demonstrated successful access in all subjects with no device‐related adverse events or device malfunctions. Fecobionics was able to record anorectal pressures, diameters, orientation, bending, and shape and the graphical user interface illustrated the integration of the data and numerous parameters were derived such as the F‐RAPG and defecation velocity. The F‐RAPG did not correlate with the ARM‐RAPG and the Fecobionics push data. It was not a specific aim to compare with the original Fecobionics device. The basic design of Fecobionics was not changed but features were added in the upgraded device. Furthermore, studies were done on an Asian population in the prior studies (Gregersen et al., 2019) whereas this study was done on a representative population of Californians and with differences in protocols. However, comparing basic data between the previous (Gregersen et al., 2019) and this study reveals that data are in the same range, for example, the expulsion duration, number of defecatory contractions, and the frequency of paradoxical contraction. Moreover, we did not aim to study patients with defecatory disorders since we need to understand the new metrics in a physiological context before moving on to clinical trials. Future clinical trials will require investigational device exemption (IDE) or 510(k) FDA approval in US and similar approvals in most other developed countries.

Methodological aspects of the device including pressure recordings and bending angle have been described before (Kassab et al., 2021; Sun et al., 2019, 2020). The major advancement of the new technology were increased data sampling frequency, adding impedance planimetric measurements, and a novel graphical user interface in a wireless probe. The comparison with conventional technologies was limited to evacuation duration and RAPG. The major limitation of this study is the small sample size; for example, it was too small to compare gender and subgroups. Some statistical comparisons may have been significant if a larger population had been studied. Most statistical comparisons showed significant differences and associations, however, which was due to the use of paired comparisons. The change in bending angle during push and straining on average showed expected trends. However, the comparisons were not significant. This is likely due to the low number of subjects, difference in exact location of the device and that the subjects in some cases did not manage to do the procedures well. Finally, the major aim was to illustrate integrated data graphically and several examples are provided in the figures. The subjects were included based on interviews and questionnaires. During the recruitment process, six subjects were excluded because they had questionnaire scores that marginally exceeded the limits. These subjects will be reported in future publications. The included subjects showed pressure profiles similar to previous Fecobionics studies and only one subject had a paradoxical contraction. However, in the 14 subjects, ARM and BET showed abnormality in 12 and 6 subjects, respectively. Normal evacuation is best done in privacy but one challenge is to assess accurately the evacuation duration. A major advantage of Fecobionics is that it is easy to measure when evacuation starts and ends from the pressure tracings. Other aspects of differences between Fecobionics and ARM‐BET have been discussed previously (Chen et al., 2020; Gregersen et al., 2019, 2020; Gregersen, Chen, et al., 2021; Gregersen, Sun, et al., 2021; Kassab et al., 2021).

Current tests have been criticized for not reflecting defecatory physiology. BET, ARM, defecography, and dynamic pelvic MRI are indirect surrogates for the act of defecation, and provide incomplete and often conflicting information (Palit et al., 2016). Though tests such as BET has proven to be a simple and useful screening test to identify constipated patients (Minguiz et al., 2004), simulated defecation is an important part of the ARM protocol, and other studies have shown good agreement between MR defecography and HR‐HRM for diagnosis and differentiation of normal evacuation and dyssynergia (Heinrich et al., 2015), publications have in general revealed that the results of these tests show low correlation with symptoms and treatment outcomes and often dyssynergic patters are recorded in normal subjects (Coss‐Adame et al., 2015; Grossi et al., 2016). The problem with most tests is that they do not provide detailed physiological data during evacuation, reflecting the dynamics of the defecation process. One specific problem with BET is that the defecation time is reported by the subjects studied and hence are subjective compared to the pressure‐based Fecobionics measure of defecation time. The Fecobionics data show that the subjectively reported expulsion time is different from the real defecation time. Differences in defecation time between the two techniques may be influenced by the different distension approaches (50 ml vs. urge‐to defecation volume). In any case, the fact that six subjects failed to defecate BET (consistent with outlet dysfunction) calls for better standardization of commercially available balloons. Fecobionics seems better in excluding abnormal defecation patterns since all normal subjects could defecate the device within a short time.

In summary, a new approach using innovative devices that can provide real‐time, quantitative, and mechanistic insights by simulating defecation through multi‐dimensional measurements of pressure profiles, deformability, bending, and topographic changes is warranted.

### 4.1 Physiological aspects

The ability to measure integrated data is important for understanding organ function. This paper presents a wealth of integrated data. We display and analyze data to a certain level including the rear‐front pressure (preload‐afterload) plot (Gregersen, 2020; Gregersen et al., 2019; Kassab et al., 2021). However, much more can be done in the future with data analysis and introduction of mathematical models to better understand defecation. Some of the work has started; for example, accelerometer data from the MPUs in Fecobionics were used to compute displacement, which is key for assessing perineal descent (Zhuang et al., 2021). Future studies will demonstrate if the endpoints have clinical value. Previous publications with Fecobionics have demonstrated significantly different patterns in patients with FI and obstructed defecation. It is anticipated that the additional data generated by the upgraded Fecobionics will provide new possibilities based on rear‐front pressure plots and metrics such as the RAPG and defecation indices (Chen et al., 2021b; Gregersen, Chen, et al., 2021).

Successful defecation depends on numerous factors including the ability to generate high intra‐abdominal pressure and to relax the anal sphincter and the puborectalis muscle. Previous experimental and analytical studies have emphasized the importance of pressure difference; that is, the RAPG used in ARM studies. The rectoanal gradient is defined as rectal pressure minus residual anal pressure, hence a positive gradient indicates a normal defecatory maneuver using the standard approach. However, when using high‐resolution and high‐density ARM, this gradient is negative in a large proportion of normal subjects (Grossi et al., 2016; Li et al., 2013; Noelting et al., 2012) particularly when the maneuver is performed with the balloon deflated. Therefore, it does not seem to be a good index to discriminate between normal individuals and patients with dyssynergic defecation. Furthermore, rectoanal gradient results are dependent on the position adopted during the evacuation maneuver (lateral sideward or sitting), and the balloon inflation status (Rao et al., 2006; Ratuapli et al., 2013). It is not clear why the RAPG is negative in normal subjects. Sauter and coworkers (Takeuchi et al., 2003) hypothesized that simulated defecation may drive the recording catheter against the wall of the anal canal, producing “contact pressure.” It is conceivable that the complete relaxation of the anal sphincter seen on ARM is an artifact caused by movement of the pressure sensors out of the anal sphincter during abdominal straining. In one study, a significant displacement of the catheter during simulated evacuation was observed in 55% of overall normal individuals, which may tend to exaggerate anal relaxation during this maneuver (Sauter et al., 2014). This may not affect high‐resolution ARM because the topographic display makes it easier for the operator to minimize probe movement during the procedure. In this study, we computed a similar parameter called the F‐RAPG. It was based on real evacuation data and provided positive values as expected. There was no association between the F‐RAPG and the ARM‐RAPG or Fecobionics pressure difference during the push procedure. This questions the use of the push procedure for evaluation of pressure differentials. Push procedures with ARM and Fecobionics may still have a value in biofeedback training. Many factors may influence the difference between the F‐RAPG and the ARM‐RAPG including the stiffness of the devices, axial vs radial pressure measurements, simulated vs real evacuation, et cetera.

### 4.2 Future aspects and conclusions

We demonstrated successful visualization of integrated mechanical data in normal human subjects. The device and procedures were safe. Fecobionics provides several improvements to current anorectal functional assessment technologies, including mechanical properties that mimic stool, objective electronic measurement of the anorectal angle, and pressure measurements in the direction of the trajectory. Fecobionics has significant potential to shift the current paradigm since it is a simulated stool that provides new metrics not simultaneously assessed with current technologies. New metrics include the F‐RAPG and bending angle opens up for calculation of defecation indices (Chen et al., 2021b; Kassab et al., 2021) based on combined pressures and geometric data. Furthermore, we question the ARM‐RAPG procedure due to the negative values and the lack of correlation with the F‐RAPG, which was done during defecatory conditions rather than during push. Future larger‐scale studies are needed to establish normal ranges and to shed more light on defecatory mechanisms in health and disease.

## AUTHOR CONTRIBUTIONS

Hans Gregersenand GSK designed the study, obtained funding, and advised the experimental work during the study period. Yanmin Wang, Mengjun Wang, Xiaomei Guo, Fred Field, and Hans Gregersenparticipated in the experiments. Data analysis was conducted by Yanmin Wang, Kar Man Lo, and Hans Gregersenand interpreted by all authors. Hans Gregersenand Kar Man Lodrafted the manuscript. All authors had access to the data, revised the manuscript, and approved the final version for submission. Hans Gregersenis the guarantor of the article.

## CONFLICT OF INTEREST

Gregersen and Kassab have filed patent applications on the Fecobionics technology. No other conflicts of interest were noted.

## Supporting information



Video S1
